# Extensive Growth of an Anaplastic Meningioma

**DOI:** 10.1155/2013/527184

**Published:** 2013-10-31

**Authors:** Hajrullah Ahmeti, Homajoun Maslehaty, Athanasios K. Petridis, Alexandros Doukas, Mehran Mahvash, Harald Barth, H. M. Mehdorn

**Affiliations:** Department of Neurosurgery, University Hospitals Schleswig-Holstein, Campus Kiel, Arnold-Heller Street 3, 24105 Kiel, Germany

## Abstract

We present the case of a 30-year-old male patient with an almost complete destruction of the calvarial bone through an anaplastic meningioma diagnosed in line with dizziness. Neuroimaging revealed an extensive growing, contrast enhancing lesion expanding at the supra- and infratentorial convexity, infiltrating and destroying large parts of the skull, and infiltrating the skin. Due to progressive ataxia and dysarthria with proven tumor growth in the posterior fossa in the continuing course, parts of the tumor were resected. A surgical procedure with the aim of complete tumor resection in a curative manner was not possible. Six months after the first operation, due to a new tumor progression, most extensive tumor resection was performed. Due to the aggressive and destructive growth with a high rate of recurrence and tendency of metastases, anaplastic meningiomas can be termed as malignant tumors. The extrinsic growth masks the tumor until they reach a size, which makes these tumors almost unresectable. In the best case scenarios, the five-year survival is about 50%. With the presented case, we would like to show the aggressive behavior of anaplastic meningiomas in a very illustrative way. Chemotherapy, radiotherapy, and surgery reach their limits in this tumor entity.

## 1. Introduction

Meningiomas are usually slowly growing tumors, arising from the arachnoid, and account for 13 to 26% of all intracranial tumors [1]. Parasagittal meningiomas are most frequently seen in up to 20.8%, followed by localisation at the convexity in up to 15.2%, tuberculum sellae in 12.9%, and sphenoid wing in 11.9% [2]. Meningiomas are classified by the World Health Organisation (WHO) into three grades [3–5]. Grade 1 meningiomas are benign lesions and are found in 90% of all cases. Grade 2 meningiomas—also called atypical meningiomas—occur in 5 to 7% of all cases and have a semi-benign behaviour with a higher rate of recurrence compared to grade 1 lesions. Anaplastic meningiomas are diagnosed in only 1 to 3% and are very aggressively growing lesions with the highest recurrence rates after surgical removal of 50% to 75% [3, 6–8]. 

Despite the wellknown entity of anaplastic meningiomas, physicians are surprised from time to time, facing bizarre manifestations of this disease. Concerning this, we present an exceptional case of a young man with extensive growth of an anaplastic meningioma located supra- and infratentorially with huge destruction of the skull. 

## 2. Case Presentation

**Figure 1:**
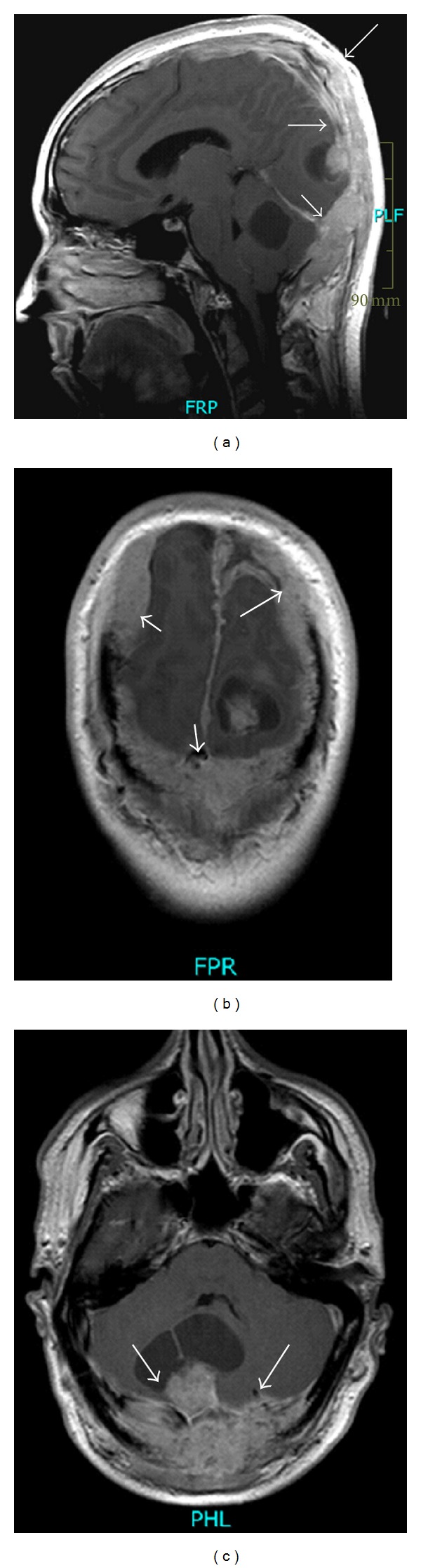
Cranial MRI with gadolinium. (a) Sagittal T1-weighted MRI demonstrates contrast enhancing tumor expanding from frontal to suboccipital. (b) Coronar T1-weighted MRI shows the fronto-parietal expansion of the tumor, cystic lesion left occipital, and the tumor mass suboccipital. (c) Axial T1-weighted indicates the tumor and cystic lesion suboccipital with compression of the forth ventricle.

**Figure 2:**
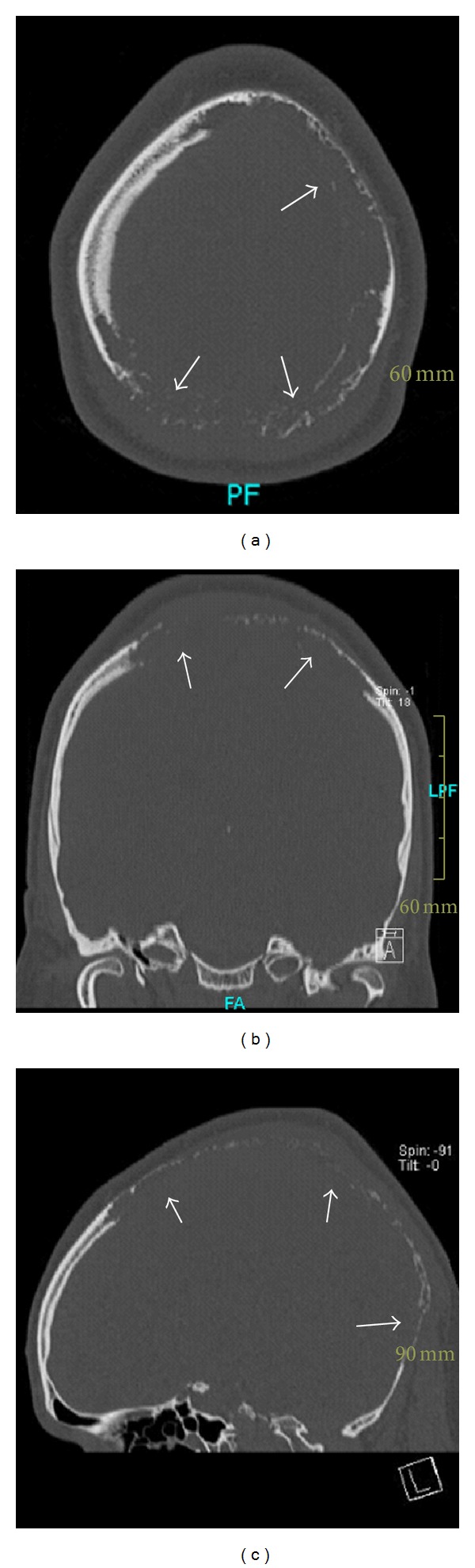
Cranial CT scan. (a) Axial view. (b, c) Coronar and sagittal reconstruction views. The cranial CT scan shows a massive bon destruction.

**Figure 3:**
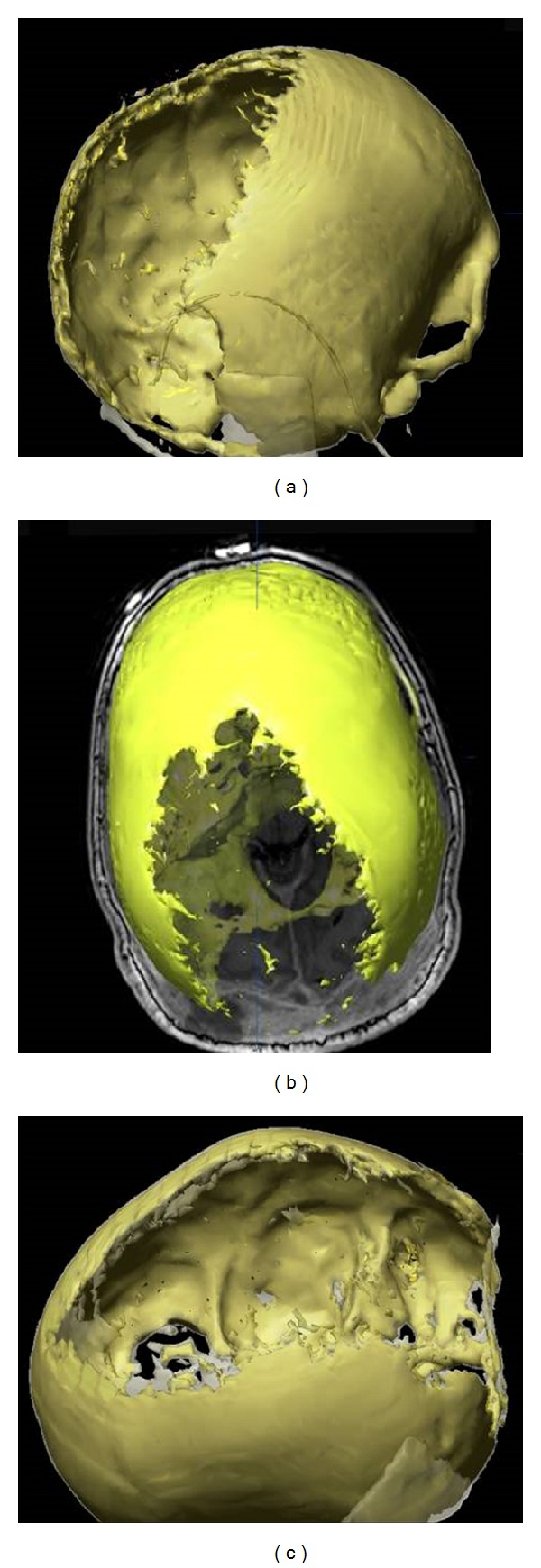
Three dimensional reconstructions with Brainlab. (a) Right lateral view. (b) View from above. (c) Left lateral view. The three dimensional reconstructions indicate clearly the dimension of bon destruction.

A 30-year-old male patient presented with pulsation of the skull and dizziness during bending down. Neurological examination revealed a dysdiadochokinesia on both sides, no further neurological deficits. The scalp was soft. A bone resistance was not palpable. Cranial magnetic resonance imaging (MRI) showed an extensive growing, contrast enhancing lesion expanding at the supra- and infratentorial convexity, infiltrating and destroying large parts of the skull, and infiltrating the skin (Figure 1). CT scanning and 3D-reconstruction images stress out the massive destruction of the calvarian bone (Figures 2 and 3).

A surgical procedure with the aim of complete tumor resection in a curative manner was not possible, so that a digital subtraction angiography (DSA) was done to identify the arterial blood supply of the tumor. The tumor was fed by branches of both internal and external carotid arteries and additionally by the right vertebral artery. The feeding branches were embolised to induce decrease of tumor size and growth. The patient was discharged and was followed closely in our out-patient department.

**Figure 4:**
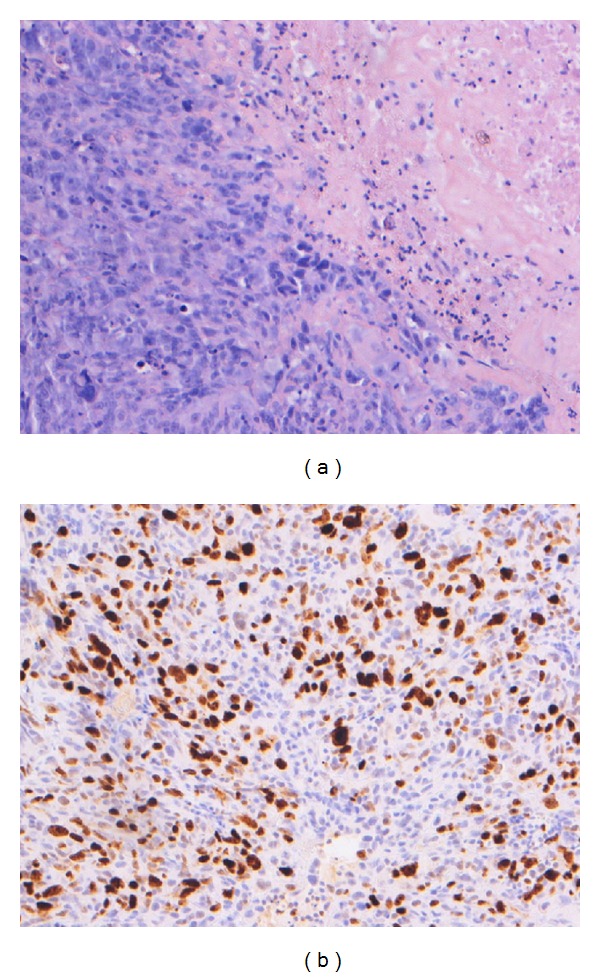
Histopathological examination. (a) HE staining reveals a meningeal tumor with high cell density, tall polymorph nucleuses, and many mitoses. (b) MIB-1 immunohistochemical staining shows a high proliferation index, 60%.

Two months later, the patient presented with progressive ataxia and dysarthria. On examination, we discovered circumscribed skin necrosis of the skull in consequence of embolization of the feeding arteries and the skin infiltration of the tumor. Despite previous embolization, MRI showed marked increase of the infratentorially localized tumor with cystic components with compression of the fourth ventricle and the brainstem. In consideration of the new findings, we resected parts of the tumor located in the posterior fossa and at the occipital convexity via an extended sub-occipital approach. During surgery, the tumor was located in the epidural space with diffuse infiltration of the bone and the skin. Histopathological work-up revealed an anaplastic meningioma WHO grade 3 with high cell density, tall polymorph nuclei and high mitotic rate with a high proliferation index of 60% (Figure 4). Molecular genetic examination of the tumor tissue showed no chromosomal aberrations. The patient recovered well from surgery and was discharged without neurological deterioration. 

**Figure 5:**
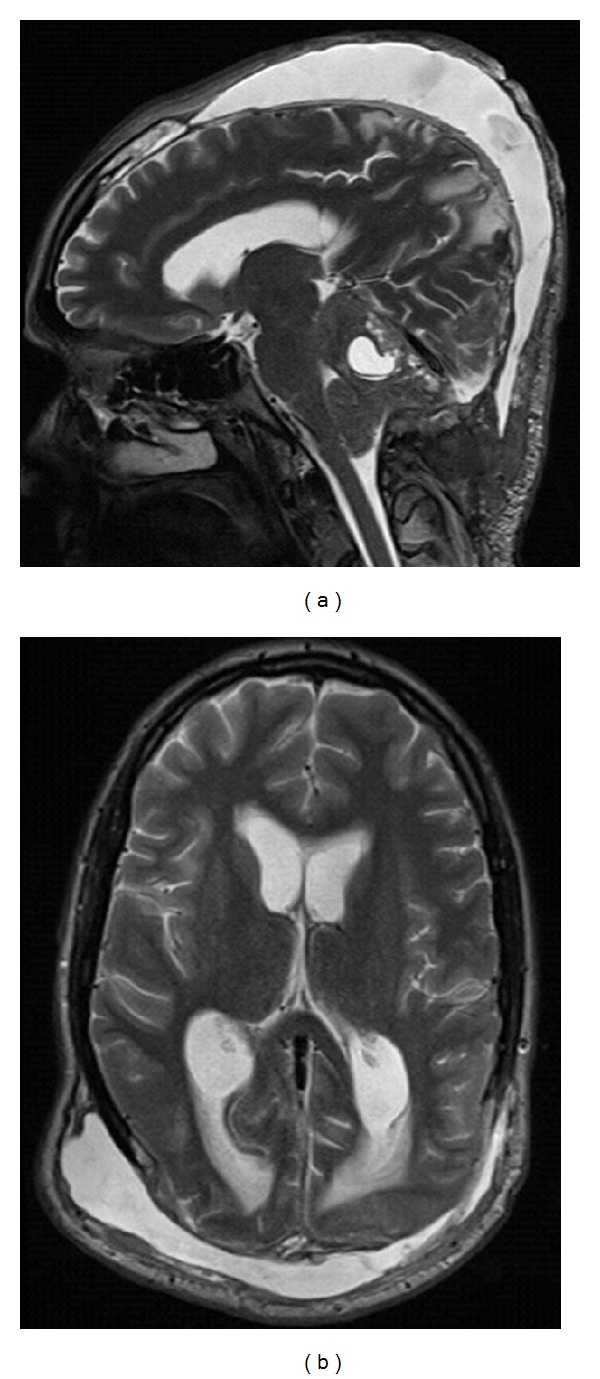
Cranial T2-wighted MRI. (a) Sagittal. (b) Axial. The cranial MRI shows a CSF circulatory disturbance with subcutaneous CSF accumulation.

**Figure 6:**
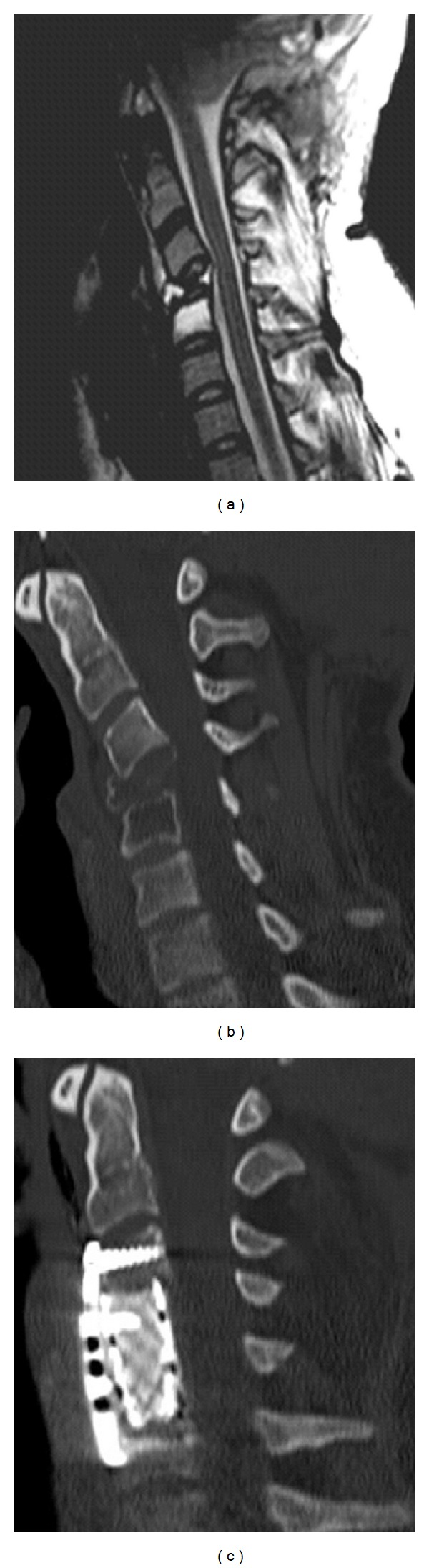
MRI and CT of cervical spine. (a) Preoperative MRI sagittal view. (b) CT sagittal view preoperative. (c) Preoperative CT sagittal view. (a) and (b) demonstrate the pathological fracture of the cervical vertebral bodies 4 and 5. (c) illustrates the postoperative result after corpectomy of the cervical vertebral bodies 4 and 5.

Six months after the first operation in a followup MRI scan, a clear tumor progression was found. We performed a new operation with the most extensive tumor resection. In the further course, the patient developed a subcutaneous CSF accumulation in the region of surgical approach. A cranial MRI scan showed CSF circulatory disturbance (Figure 5). For further clarification of new-onset findings, a MRI scan of the spinal axis was initiated. The MRI scan showed metastasis of the cervical spine with a pathological fracture of the 4th and 5th vertebral bodies. Foremost, a corporectomy of the cervical vertebral bodies 4 and 5 accompanied with anterior fusion with plate and screws was conducted (Figure 6). A ventriculoperitoneal shunt was afterwards implanted. The cervical spine was then irradiated. The patient died 17 months after diagnosis of tumor.

## 3. Discussion

Anaplastic meningiomas are aggressive and rapid growing tumors with frequent infiltration of the surrounding tissue. De novo arising is described, as well as secondary development from benign and atypical meningiomas [1]. Patients with anaplastic meningiomas are significantly younger compared to those with grade 1 or 2 tumors, with a slightly higher predominance for the male gender (male to female ratio: 1 : 0.9) [2, 3]. Frequent clinical symptoms have to be considered in view of cerebral mass effect and elevated intracranial pressure, such as headaches, nausea, and vomiting [2]. Focal neurological impairments such as cranial nerve disorders are dependent of tumor localisation and are not predictive for anaplastic meningiomas. 

As the most important prognostic factor, the treatment of choice should aim at complete microsurgical tumor resection (Simpson °I) followed by radiotherapy, which increase the recurrence free interval to the median of 34.1 months [2]. The five-year survival in complete resected anaplastic meningiomas is estimated with 28%. Complete resection and radiotherapy increases the five year survival towards 57% [4].

However, complete tumor resection might be difficult in complex skull base meningiomas or impossible in widespread tumors because of the surgical accessibility, just as in the presented case.

There are no effective adjuvant chemotherapy agents for anaplastic meningiomas available [5]. Applications of cyclophosphamide, Adriamycin, and vincristine are described but without satisfying results [6]. Thus, the prognosis of anaplastic meningiomas in spite of complete tumor resection and adjuvant radiotherapy remains poor, due to a high rate of recurrence and metastases [5].

Kim and coworkers have defined different factors and analyzed their impact on recurrence in anaplastic meningiomas. The factors concern clinical parameters, histopathological findings, WHO grading, and cytogenetic aspects [7, 8]. 

Our presented case illustrates a worse clinical constellation because of the limited surgical treatment possibilities due to the extensive growth of the tumor. Moreover, embolization of the feeding vessels failed to reduce the tumor size. Furthermore, the presence of many negative predictive factors leads to an unfavourable prognosis.
